# Epilepsia Partialis Continua in a Post-stroke Patient: A Case Report

**DOI:** 10.7759/cureus.98013

**Published:** 2025-11-28

**Authors:** Adam Zoubi, Deobrat Mallick

**Affiliations:** 1 Internal Medicine, Christus Spohn Hospital, Corpus Christi, USA

**Keywords:** continuous jerky movement, eeg-negative seizures, epilepsia partialis continua (epc), epilepsy after cerebrovascular accident, focal status epilepticus, post-stroke epilepsy

## Abstract

We report a case of continuous, involuntary jerky movements involving the right upper extremity in a patient with a prior cerebrovascular accident affecting the same side years prior to the discussed admission. The patient had persistent regular jerky movements, without loss of consciousness or generalized convulsions, which raised suspicion for epilepsia partialis continua (EPC), a rare form of focal status epilepticus characterized by continuous motor seizures affecting one part of the body. Recognition of EPC is critical and requires high clinical suspicion, as imaging (CT, MRI) or EEG may not show any specific findings or pathologies. This can lead to delays in appropriate antiepileptic management, noting that EPC requires polytherapy of multiple antiepileptic drugs while addressing the leading cause. This case highlights the importance of maintaining a high index of suspicion for EPC in a patient with focal motor activity following stroke and underscores the need for prompt diagnosis and treatment to prevent further neuronal injury.

## Introduction

Epilepsia partialis continua (EPC) is a rare form of focal status epilepticus characterized by continuous or near continuous, repetitive muscle jerks limited to one part of the body, often persisting for hours to days without impairment of consciousness [1]. It is most commonly associated with structural brain lesions such as stroke, cortical dysplasia, infections, or autoimmune and metabolic disorders. Because imaging and electroencephalographic findings may be subtle or nonspecific [1], EPC can easily be misdiagnosed as spasticity [2], clonus [3], or post-stroke movement disorders, including different types of post-stroke seizures [4,5]. Early recognition and aggressive management are essential to prevent further neuronal damage and functional decline. We present the case of a patient with multiple comorbidities, including a history of cerebrovascular accident, who developed persistent, involuntary jerky movements of the right upper extremity, ultimately diagnosed and treated as EPC.

## Case presentation

A 54-year-old male with past medical history of cerebrovascular accident (old right frontal parietal region six years prior to the admission discussed, and more recent infarction within the left frontal and parietal lobes within the anterior cerebral artery (ACA) distribution three years prior to admission discussed in this case) with residual right hemiparesis, chronic kidney disease stage IIIb (CKD), diabetes mellitus type II, diabetic retinopathy, congestive heart failure, hyperlipidemia, left retinal detachment (blindness of the left eye), depression, obstructive sleep apnea and hypertension presented from nursing home where he lives for confusion compared with his orientation baseline.

Subjectively, the patient did not report any concerns or complaints, denying any pain, shortness of breath, chills, cold sweats, or fevers. The initial physical exam showed right-sided weakness compared to the left side, although strength is 5/5. Otherwise, the physical exam was unremarkable. However, the investigative laboratory workup includes basic chemistry in Table 1, and venous blood gas (VBG) in Table 2. Complete blood count (CBC) was unremarkable and all within normal limits.

**Table 1:** Basic Metabolic Panel

Test	Result	Reference Range/Normal Values
Creatinine	5.2 mg/dL	0.6-1.3 mg/dL
Estimated glomerular filtration rate (eGFR)	12 mL/min/1.73 m^2^	≥60 mL/min/1.73 m^2^
Blood urea nitrogen (BUN)	83 mg/dL	7-20 mg/dL
Sodium (Na^+^)	143 mEq/L	135-145 mEq/L
Chloride (Cl^-^)	112 mEq/L	96-106 mEq/L
Potassium (K^+^)	3.1 mEq/L	3.5-5.1 mEq/L

**Table 2:** Venous blood gas

Test	Result	Reference Range/Normal Values
CO_2_ (pCO_2_)	44.6 mmHg	40-50 mmHg (venous)
Bicarbonate (HCO_3_^-^)	15.4 mEq/L	22-28 mEq/L
pH	7.16	7.31-7.41 (venous)

**Figure 1:**
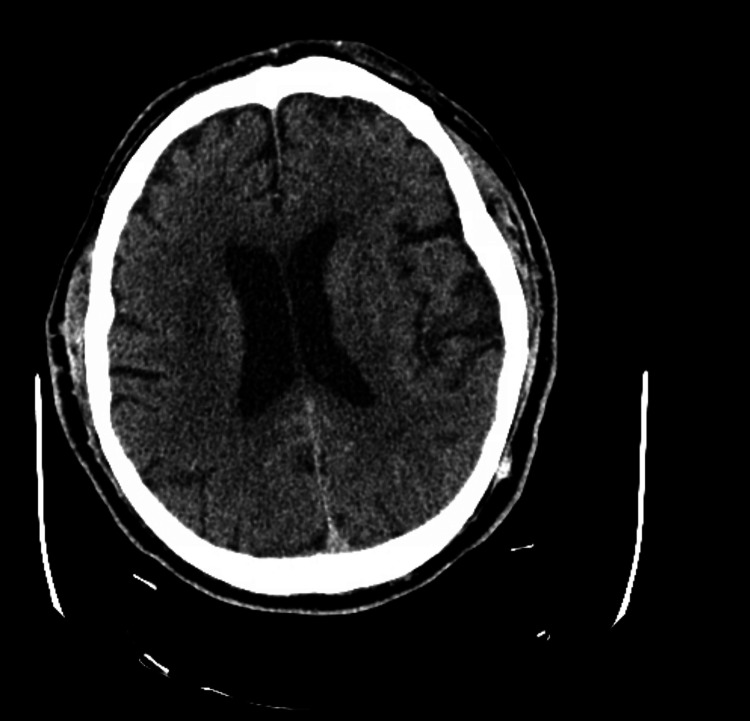
CT head without acute findings No acute intracranial hemorrhage is identified. There is no mass, mass effect, midline shift, or abnormal extra-axial fluid collection. Stable encephalomalacia is noted in the superior left frontoparietal lobes. No CT evidence of acute ischemia is seen. Scattered hypodensities in the supratentorial white matter are nonspecific but likely represent chronic small-vessel ischemic changes. The ventricular system and sulcal spaces are enlarged but proportionate, consistent with diffuse cerebral atrophy. The basilar cisterns are patent. The visualized orbits and globes show no acute abnormality, and no skull fracture is identified.

The CT head without contrast didn't show any acute changes, as shown in Figure 1.

**Figure 2:**
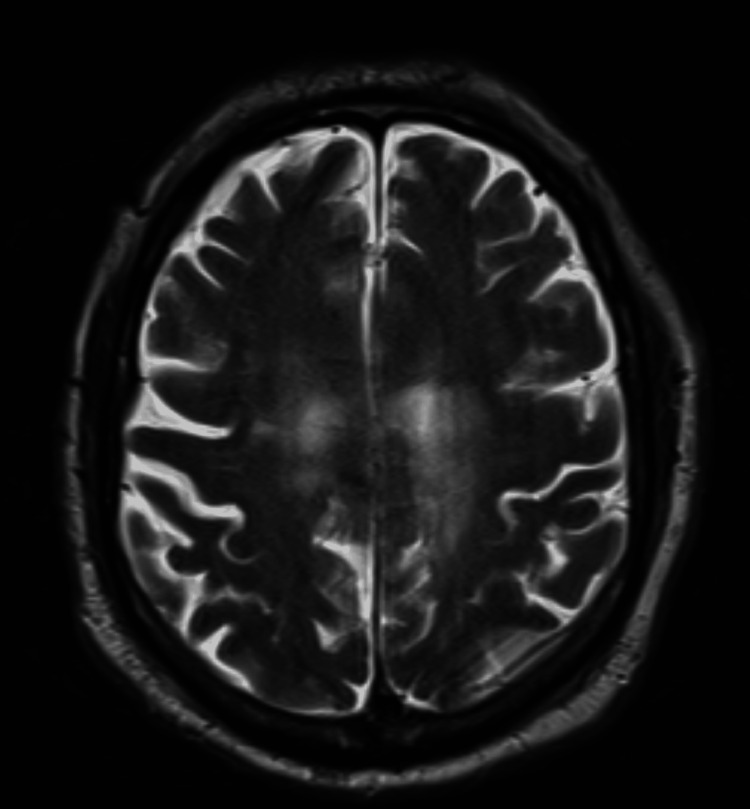
MRI brain without contrast MRI shows no acute infarct, hematoma, mass, extra-axial collection, or hydrocephalus. There is a chronic infarct in the paramedian posterior left superior frontal gyrus, extending into the pre- and postcentral gyri. Mild chronic microvascular ischemic changes are present. Foci of GRE dephasing in the left lentiform nucleus and right cerebellum suggest sequelae of prior hypertensive ischemia. There is generalized parenchymal volume loss. Normal flow-voids are observed in the major intracranial vessels. The sellar and pineal regions are unremarkable, and the craniovertebral junction is intact. GRE: gradient-recalled echo

On the seventh day of admission, the patient began experiencing continuous, unintentional, regular small jerky movements of the right upper extremity happening every two to three seconds, causing mild wrist extension and bicep flexion, during which the patient did not lose or have any altered consciousness. The patient was aware of the symptoms, and this had never happened to him before. These symptoms lasted six to eight hours each time they happened. He did not bite his tongue or lose bladder/bowel control, nor has he had any generalized tonic clonic movement presently or in the past. A 2mg of Ativan IV was given, which partially improved the symptoms, before the involuntary movements recurred again after an hour. During that time, an MRI, as shown in Figure 2, was ordered and showed chronic ischemic changes without any acute-appearing imaging abnormalities.

Neurology services were consulted, and stat continuous electroencephalography (cEEG) was ordered. Initial STAT cEEG interpretation duration was 60 minutes between 1600 and 1700. EEG was performed utilizing the standard international 10-20 system of electrode placement, and a channel electrocardiogram was monitored. Data were obtained and interpreted utilizing referential montage recording, with reformatting to longitudinal, transverse bipolar, and referential montages as necessary for interpretation. State monitored were awake, drowsy, and asleep.

Stat interpretation of a long-term video EEG showed changes specific to and/or consistent with the states of waking, drowsiness, and sleep. Drowsiness and sleep predominated, with little representation of the most normal waking state, which itself was abnormal due to the slowing of the posterior dominant rhythm.

The waking background was mildly abnormal: amplitude was normal at 20-40 uV and continuous, asymmetric (slower on left), but background organization was abnormal, with anterior-posterior organization mildly subnormal and posterior dominant rhythm during waking slowed (~6 Hz) and only briefly present. There was frequent, very brief to brief bilateral left > right shifting irregular theta slowing beyond what would be typical for the state. No clear EEG correlate was seen with the observed twitching movements. No seizures or other epileptiform or focal findings were identified. Overall, these findings are consistent with mild bilateral (left > right) cerebral dysfunction without cortical irritability.

The second interpretation duration was 12 hours between 0100 and 1300 on the day after the initial stat read, using the same technical description mentioned above. This study was predominantly in the sleep state, with brief arousals characterized by diffuse, predominantly theta activity. A well-formed posterior dominant rhythm was not seen. Recording during the awake state was noted more in the latter part of the recording. Stage 2 sleep was characterized by symmetric sleep spindles. The background was asymmetric, and near-continuous focal delta/theta slowing was noted in the left hemisphere. No epileptiform discharge or seizures were seen. This abnormal EEG is consistent with focal cerebral dysfunction/structural lesion in the left hemisphere, along with mild diffuse encephalopathy that is nonspecific in etiology.

The third interpretation duration was three hours, right after the second one mentioned above, which lasted between 1300 and 1600. EEG description: During the maximally awake state, a well-formed posterior dominant rhythm was not seen. The background appeared asymmetric with diffuse predominant theta activity, and a near-continuous focal delta/theta slowing was noted in the left hemisphere. No clear epileptiform discharge or seizures were seen. A push-button event was noted at 1507; on video, there were right-hand twitching movements, with no clear EEG ictal pattern. Clinical correlation: This was an abnormal EEG, consistent with focal cerebral dysfunction/structural lesion in the left hemisphere and mild diffuse encephalopathy, non-specific in etiology. No clear epileptiform discharge or seizures were seen. A paroxysmal event was captured (right-hand twitching), with no clear EEG ictal pattern.

Initially, dialysis disequilibrium syndrome (DDS)-induced seizure following hemodialysis (HD) was considered. However, due to the delayed onset after initiating dialysis and the focal seizures limited to the right upper extremity, instead of the tonic-clonic seizures most commonly seen in DDS, findings on cEEG all made DDS less likely. Clinical and laboratory workup was negative for toxic metabolic etiologies, without any reported infection or toxicity. EPC was suspected, and the patient was initially started on a loading dose of IV levetiracetam 1.5g, and then maintenance renal dosing of PO 500mg BID with an additional 250mg dose post dialysis, as well as lacosamide 100mg BID, with a plan for frequent anti-epileptic medication (AED) level monitoring by a nephrologist. The patient tolerated these medications well without noticeable or reported side effects. His jerky movements progressively shortened in duration and amplitude; two days prior to discharge, the duration of these episodes was no longer than one hour and were subjectively and objectively milder in intensity. Last day prior to discharge, he was symptom-free, and the patient was discharged with nephrology and neurology outpatient follow-up.

## Discussion

The case illustrates a clinical presentation of EPC, a continuous, regular, focal, jerky movement of the right upper extremity with preserved awareness, in a patient with prior strokes affecting the contralateral (left) hemisphere. The significance of this case lies in three diagnostic and management challenges that may frequently be encountered at the bedside: EEG can be non-diagnostic despite unequivocal clinical semiology [1,6]. Imaging may be unchanged, offering possible etiologies that could have led to EPC [1]. In this case in particular, metabolic and dialysis-related factors can cloud attribution, especially in patients with advanced kidney disease undergoing HD for the first time.

Together, these features risk mislabeling EPC as post-stroke spasticity or clonus, metabolic myoclonus, or DDS, thereby delaying treatment. Recognizing EPC as a clinical diagnosis - even without EEG or imaging confirmation, which did not exclude EPC - was pivotal in this case.

EEG limitations in EPC

Scalp EEG has limited sensitivity for focal aware motor seizures, which may sometimes appear as spikes, sharp waves, or periodic lateralized epileptiform discharges (PLEDs) with slow-wave activity. As in our case and the EEG findings described above, the study may show asymmetrical background slowing, while prominent electromyographic artifacts during jerks can render scalp recordings “seizure-negative” even when clinical seizures are ongoing. This underscores that a normal or nonspecific EEG cannot exclude EPC, making the diagnosis even more challenging [6].

Imaging

Neuroimaging in EPC often reflects the underlying structural etiology [1] rather than EPC-specific change. Scans may be unchanged from baseline or show chronic lesions without acute findings (as in our case). Similar to EEG, normal or non-acute imaging changes do not lower the post-test probability of EPC when the clinical picture is supportive of EPC.

DDS as a confounder

DDS can lead to neurologic symptoms during or shortly after HD [7], particularly around initiation of dialysis or after missed sessions. Manifestations range from headache and confusion to more severe complications like seizures, coma, and death [8]. In our case, the timing (day 7 after the first HD session, not peri‑dialysis), along with purely unilateral motor jerky, regular, and continuous movements, with preserved awareness in our patient, argues against DDS as the primary cause.

In our case, several features favored EPC over other differentials: The continuous, unilateral, regular jerks with preserved awareness fit EPC and map anatomically to the contralateral (left) perirolandic/medial frontal cortex seen in old and repeat MRI, consistent with this patient’s prior left ACA territory infarct and the left-greater-than‑right background slowing on EEG. The cEEG showed left predominant focal slowing without ictal patterns, a known limitation in EPC, where small or deep cortical sources and movement artifacts can mask ictal rhythms on scalp leads. Thus, a non‑confirmatory EEG is compatible with EPC and should not delay treatment when the clinical picture is compelling.

Ruling out DDS

The absence of peri‑dialysis onset, the focal nature of movements, and the lack of generalized tonic‑clonic activity reduce the likelihood of DDS. DDS more typically presents near the time of dialysis with global symptoms, and when seizures occur, they are often generalized rather than strictly focal events. Lastly, there were no signs of sepsis, infection, or other metabolic or toxic etiologies to explain the presentation.

Therapeutic considerations

As mentioned in our case, benzodiazepines often do not achieve sustainable control [1,9], necessitating polytherapy with AEDs. Levetiracetam may be the first choice for managing EPC related to ischemic stroke [10], and in our case, it was selected due to the patient’s history of CKD progression requiring dialysis. Adding multiple AEDs is sometimes necessary - such as phenytoin, valproate, or lacosamide - but initiating multiple agents requires close monitoring because of the increased risk of adverse effects [9].

This case reinforces three important practice points. First, EPC is a clinical diagnosis, and it should not be excluded based on a non-ictal scalp EEG or unremarkable neuroimaging when clinically suspected. Second, differentiating EPC from dialysis-related phenomena is essential; focal, persistent motor activity with preserved awareness occurring days into hospitalization is far less consistent with DDS than with EPC. Third, early and carefully adjusted treatment is crucial in patients with CKD or those on HD. AED polytherapy is often required, and both levetiracetam and lacosamide can be used effectively with appropriate renal dosing. AED use in CKD/end-stage renal disease (ESRD) carries a higher risk of adverse effects; therefore, medications should be initiated using a “low and slow” approach, titrated according to the clinical response so that the patient remains on the lowest effective dose. Regular monitoring of AED levels is essential to ensure safe and effective therapy [11].

Overall, this case corroborates the current understanding that EPC after a stroke may present with EEG-negative recordings, compelling clinicians to prioritize clinical semiology and tailor AED therapy to renal function. As in our case, a regular (non-adjusted) loading dose of levetiracetam (Keppra) can be administered initially, followed by a maintenance dose not exceeding 1g per day. To avoid drug accumulation, we started our patient on 500mg BID, which he tolerated well without reported side effects. An additional 250-500mg dose can be given after dialysis. Most importantly, AED serum levels must be monitored frequently. Similarly, no loading-dose adjustment is required for lacosamide; the maintenance dose should be approximately 75% of the usual dosing, with an additional 50% of that maintenance dose administered post-dialysis, followed by periodic monitoring of lacosamide levels. Prompt recognition and treatment likely contributed to this patient’s improvement and may help mitigate cumulative neuronal injury and disability in similar presentations.

## Conclusions

Continuous, unilateral jerky movements with preserved awareness in a post‑stroke patient should prompt strong consideration of EPC, even when cEEG is non‑ictal, and neuroimaging shows negative results or chronic changes without acute changes. In this case, the characteristic semiology, lateralization concordant with prior left‑hemispheric infarcts, and non‑diagnostic cEEG supported a clinical diagnosis of EPC over dialysis‑related phenomena. Early initiation of antiseizure polytherapy (levetiracetam and lacosamide) with appropriate dosing for renal function resulted in symptomatic improvement.

The key message is to prioritize clinical picture and suspicion over tests that can be falsely negative, differentiate EPC from mimics (including DDS, metabolic myoclonus, and post‑stroke clonus), and treat early while adjusting AED regimens for kidney function and dialysis schedules. Educators should emphasize EEG limitations and bedside observation skills. Researchers should focus on better detection methods (e.g., higher‑density/wearable EEG and artifact-resistant analytics). Timely, semiology‑driven recognition and treatment may reduce further neurological damage and morbidity in similar presentations.
